# Regression of a Myeloid Sarcoma of the Nasal Cavity With Extension to the Cheek After Radiotherapy

**DOI:** 10.7759/cureus.41273

**Published:** 2023-07-02

**Authors:** Soukaina Morchid, Nabila Sellal, Imane El Boutahiri, Safaa Regragui, Mohamed El Hfid

**Affiliations:** 1 Department of Radiotherapy, Mohammed VI University Hospital Center, Tangier, MAR; 2 Department of Hematology, Mohammed VI University Hospital Center, Tangier, MAR

**Keywords:** radiation therapy, nasal cavity, extramedullary leukemia, acute myeloid leukaemia, myeloid sarcoma

## Abstract

Myeloid sarcoma is rare and nasal chloroma is an uncommon initial manifestation of acute myeloid leukaemia. The correct diagnosis is a big challenge. In this report, we present a case of myeloid sarcoma of the nasal cavity with extension to the soft tissues of the face. A 53-year-old woman with a past medical history of thalassemia, not followed up, presented with a progressive greyish swelling in her right cheek associated with a nasal obstruction more marked on the right side and unilateral lacrimation. The diagnosis of myeloid sarcoma was based on histopathology and immunohistochemistry. Bone marrow aspiration testing revealed blasts that met the criteria for acute leukaemia. She received external radiotherapy at a total dose of 30 Gy in 15 fractions without systemic therapy, because she refused to get chemotherapy. She remained under surveillance and symptomatic treatment. The patient was examined four months after the end of the irradiation and showed a spectacular improvement in her clinical symptomatology with a clear decrease in nasal mass.

## Introduction

Myeloid sarcoma (MS), also called chloroma because of its green colour attributable to the enzyme myeloperoxidase, is defined by an extramedullary proliferation of blasts of one or more of the myeloid lineages [1], which incompletely or completely abolish the tissue armature [2]. In most cases, MS occurs at the same time in patients with an established diagnosis of acute myeloid leukaemia (AML). It can also be diagnosed de novo, as a manifestation of recurrent AML, or as a progression of anterior myelodysplastic syndrome, chronic myeloid leukaemia, and other myeloproliferative neoplasms [3]. Given its low incidence and its heterogeneity, the majority of the literature consists of case reports and small retrospective studies, which limits clinical knowledge and therapeutic management [4].

## Case presentation

**Figure 1:**
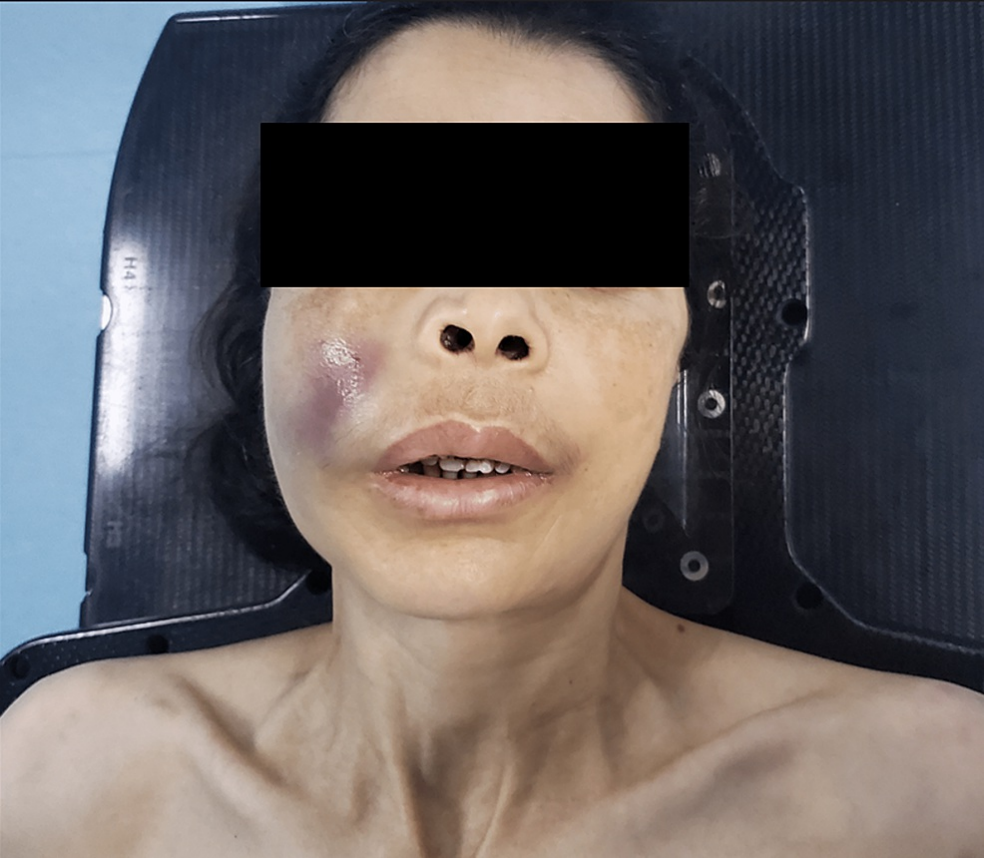
Clinical aspect before treatment

A 53-year-old woman with a medical history of thalassemia since 2006, not followed up, and a hysterectomy for uterine fibroids in 2010 presented in January 2022 with progressive greyish swelling in her right cheek, evolving for three months and associated with a nasal obstruction more marked on the right side and ipsilateral lacrimation (Figure 1), which prompted her to see a head and neck specialist. The rest of the physical examination was normal.

A computed tomography (CT) of the face reported an intranasal mass obstructing the external nares with extension to the right cheek without bone destruction. It bulged in the nasopharynx posteriorly, extended to the ethmoidal cells superiorly, and was associated with a right lateral cervical lymphadenopathy of 16 mm. A biopsy of the mass was done which showed a large number of atypical cells that were small to medium in size, with high nucleo-cytoplasmic ratio, prominent nucleoli and hyperchromatic nucleus. On immunohistochemistry, the cells expressed positivity for anti-vimentin and anti-myeloperoxidase (MPO) and were negative for CD45, CD3, CD20, CD30, creatine kinase (CK), CD 138, CD79a, CD56, P63, Synaptophysin, chromogranin, glial fibrillary acidic protein (GFAP), myogenin, Melan A, CD99, and terminal deoxynucleotidyl transferase (TdT). Thus, a diagnosis of MS was made. Complete blood count revealed pancytopenia (haemoglobin 7.1 g/dl, total leucocyte count 1780/mm^3^, and platelet count 78000/mm^3^). Peripheral smear examination showed 8% atypical cells, 15% polymorphonuclear neutrophils (PMN), 6% monocytes, and 71% lymphocytes. Bone marrow aspiration showed a cellular marrow with 75% blasts with diminished erythropoiesis and megakaryopoiesis. On immunophenotyping by flow cytometry of bone marrow, the blasts expressed MPO, CD117, and CD33, without expression of markers of immaturity or lymphoid antigens.

**Figure 2:**
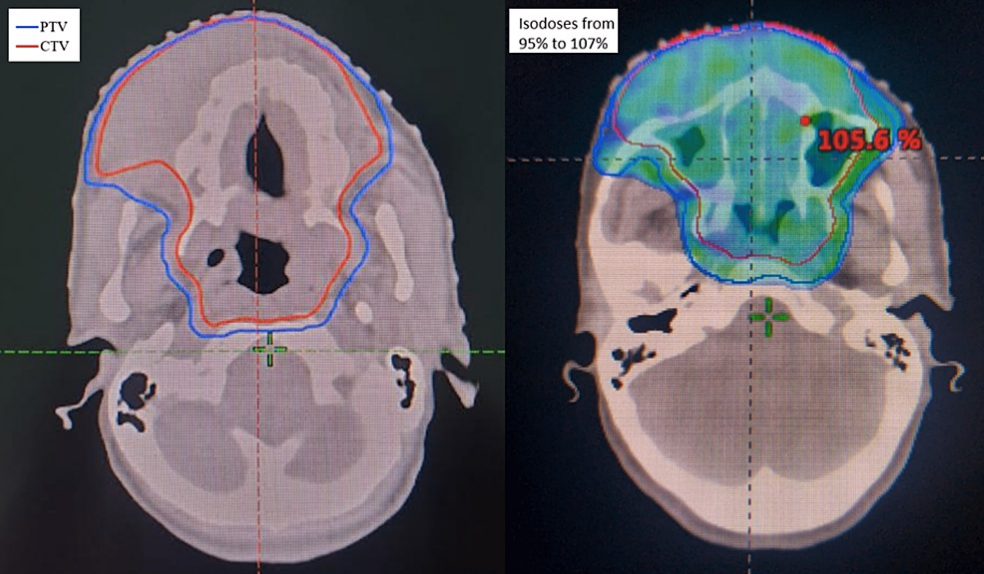
Target volume and its coverage PTV: planning target volume; CTV: clinical target volume

The patient was diagnosed with AML presenting as MS. External radiotherapy was then decided for the facial mass while including the skin. She was planned for a total dose of 30 Gray in 15 fractions (Figure 2), at a daily dose fraction of 2 Gray over three weeks, using the volumetric modulated arc therapy technique. We were able to keep the tolerance doses of organs at risk such as the optic nerves (Dmax optic nerve right (ONR)=31,3 Gy, Dmax optic nerve left (ONL)=31,8 Gy), optic chiasma (Dmax=32 Gy), brainstem (Dmax=24,6 Gy), spinal cord (Dmax = 16 Gy) and parotids (Dmean parotid right (PR)= 9,5 Gy and Dmean parotid left (PL)= 10,2 Gy) within normal limits and at the same time deliver the intended dose of radiation to the tumour site. At the end of the treatment, the patient reported an improvement in clinical symptomatology, especially nasal obstruction. The physical examination objectified a regression of the tumour process with good tolerance of radiation therapy except for a grade I radiation dermatitis. On the other hand, unfortunately, she refused to get chemotherapy. She remained under surveillance and symptomatic treatment.

**Figure 3:**
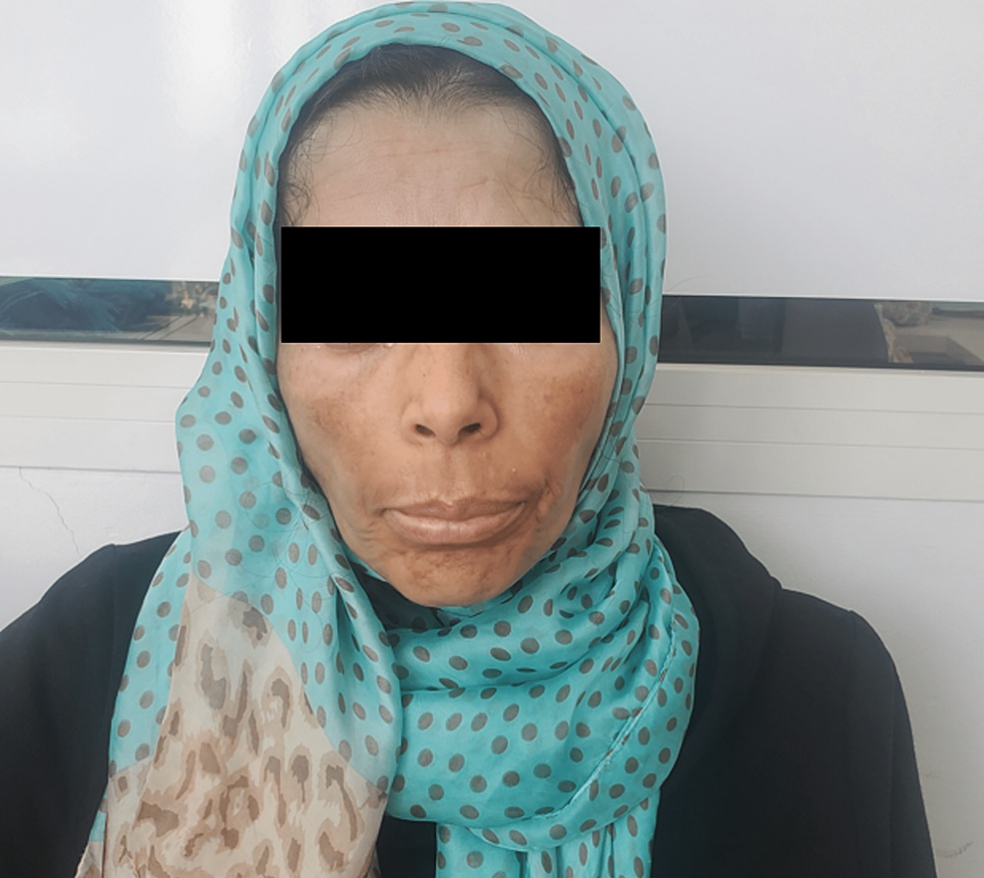
Clinical aspect four months after treatment

The follow-up after four months showed a dramatic improvement with the disappearance of the initial swelling, as well as an improvement in nasal obstruction (Figure 3).

## Discussion

AML is the result of malignant clonal expansion and differentiation arrest of immature myeloid progenitors [5]. In addition to bone marrow and peripheral blood, leukemic blasts that are characteristic of AML may also cause extramedullary ALM or MS, altering the tissue's natural architecture [3,5]. MS, which was first described in 1811, is also known as "chloroma" because of the green hue it takes on as a result of intracellular myeloperoxidase being present. Due to the tumour's variable macroscopic appearance, the tumour was renamed granulocytic sarcoma in 1966 [6]. Diagnoses of MS can be made at any age, with a small male predominance (56.1%) [3], and they can develop anywhere, but the skin, gastrointestinal tract, bone, and lymph nodes are the most often affected areas [7]. Nasal chloroma cases are quite rare, according to published reports [8].

We described a case of MS presenting as a nasal cavity mass with extension to the soft tissues of the right cheek in a 53-year-old female. In about a quarter of cases, MS is the initial sign of AML; it may precede it by months or even years. In 15-35% of cases, MS develops at the same time as AML, and in up to 50% of cases, it develops after the diagnosis of AML. It may also be the first sign of relapse in a patient who was previously treated for AML and who is still in remission [1,2]. In the present case, the discovery of MS coincides with the diagnosis of AML. The clinical presentation of MS is location-dependent, and the presenting symptoms are typically caused by the tumour's bulk effect or organ dysfunction as a result of infiltration [4]. These relatively uncommon tumours can be difficult to detect, especially if the bone marrow is unaffected. They can be mistaken for non-Hodgkin's lymphoma, Ewing's sarcoma/primitive neuroectodermal tumour, rhabdomyosarcoma, and neuroblastoma [3]. In the case of nasal mass, immunohistochemistry should be performed to rule out esthesioneuroblastoma and small-cell carcinoma [8].

Historically, nearly 75% of MS cases have been misdiagnosed. With recent improvements in diagnostic techniques, this has improved; however, 25-47% of instances still result in a misdiagnosis [3]. Imaging investigations are crucial for diagnosis, treatment planning, and therapy response monitoring [3]. CT imaging is frequently the technique of choice for MS since it typically manifests as a soft tissue mass [4]. Positron emission tomography (PET)/CT may be particularly beneficial for detecting sites of sanctuary myeloid sarcoma, monitoring response, and planning radiation therapy [3,4]. The only way to get a conclusive diagnosis, especially for patients who acquire granulocytic sarcoma without a leukaemia diagnosis, is a biopsy of the mass [6]. Eosinophilic metamyelocytes are useful diagnostic indicators in granulocytic differentiation cases, whereas monocytic neoplasms are more challenging to diagnose, necessitating the use of immunohistochemistry and auxiliary studies like flow cytometry, fluorescence in situ hybridization (FISH), cytogenetics, and molecular studies [3]. Immunohistochemically, CD68 is frequently a positive marker, followed by MPO, CD117, CD99, lysozyme, and CD34 [7].

Concerning treatment strategies, a universal agreement has not yet been established with regard to therapeutic alternatives because of the rarity of the disease and the diversity of body sites and age groups [6,8]. The exclusive local treatments of isolated MS do not seem sufficient, since in the absence of systemic treatment the disease evolves inexorably towards acute leukaemia in 88-100% of cases versus 42% when systemic treatment is delivered to the patient [9]. In addition, several studies, including those of Tsimberidou et al. [10], Imrie et al. [11], and Cunningham et al. [12] have shown an improvement in the probability of overall survival or survival without progression when a multimodal treatment was offered to the patient (more than 50% of surviving patients for a median follow-up of 25 months per compared to a median survival time of 13 months for untreated patients). In cases of isolated MS and AML patients who are resistant to systemic therapy, recurrence after bone marrow transplantation and when rapid symptom relief is needed, radiotherapy should be taken into account. Most MS patients can receive a normal dosage of 24 Gy in 12 fractions with conventional photon radiation with excellent disease management and minimal side effects [4,5]. Our patient received palliative radiotherapy, which provided rapid relief of symptoms.

## Conclusions

MS is an extramedullary event of AML and can sometimes be the only sign of the disease. It is rarely seen in the nasal cavity. Due to its unusual presentation, the correct diagnosis is difficult. Because of the disease's rarity and lack of randomized controlled studies, there is no consensus on the treatment of MS. Radiation therapy appears to provide local control of chloromas with low doses, which is the case for our patient, but it is not sufficient and must be associated with systemic therapy.
